# Supplementary material for: Maize-peanut rotational strip intercropping improves peanut growth and soil properties by optimizing microbial community diversity
Source: PeerJ. 2022 Jul 28;10:e13777. doi: 10.7717/peerj.13777 (PMC9339216; doi:10.7717/peerj.13777)
Supplement: Supplemental Information 2 — This dataset contains additional material for the article’s average temperature, rainfall, and rhizosphere soil microbe-related indicators. [file peerj-10-13777-s002.zip › Peer J_Supplemental Information/Supplementry_Maize¿Cpeanut rotational strip intercropping improved peanut growth, optimized soil properties and impacted microbial community diversity.docx]

*Article*

**Maize-peanut rotational strip intercropping improves peanut growth and soil properties by optimizing microbial community diversity**

**Yi Han^1^, Qiqi Dong^1^, Kezhao Zhang^1^, Dejian, Sha^1^, Chunji Jiang ^1^, Xu Yang^1^, Xibo Liu^1^, He Zhang^1^, Xiaoguang Wang^1^, Feng Guo^2^, Zheng Zhang^2^, Shubo Wan^2^, Xinhua Zhao^1*^, Haiqiu Yu^1*^**

^1^ College of Agronomy, Shenyang Agricultural University, Shenyang 110866, P.R.China

^2^ Biotechnology Research Center, Shandong Academy of Agricultural Sciences, Jinan, P.R.China

***** Correspondence: [xinhua_zhao@syau.edu.cn](mailto:xinhua_zhao@syau.edu.cn); Tel.: 13840010478; [yuhaiqiu@syau.edu.cn](mailto:yuhaiqiu@syau.edu.cn); Tel.:13674201361





**Figure S1.** Monthly average temperature and precipitation during the 2015-2018 growing season.


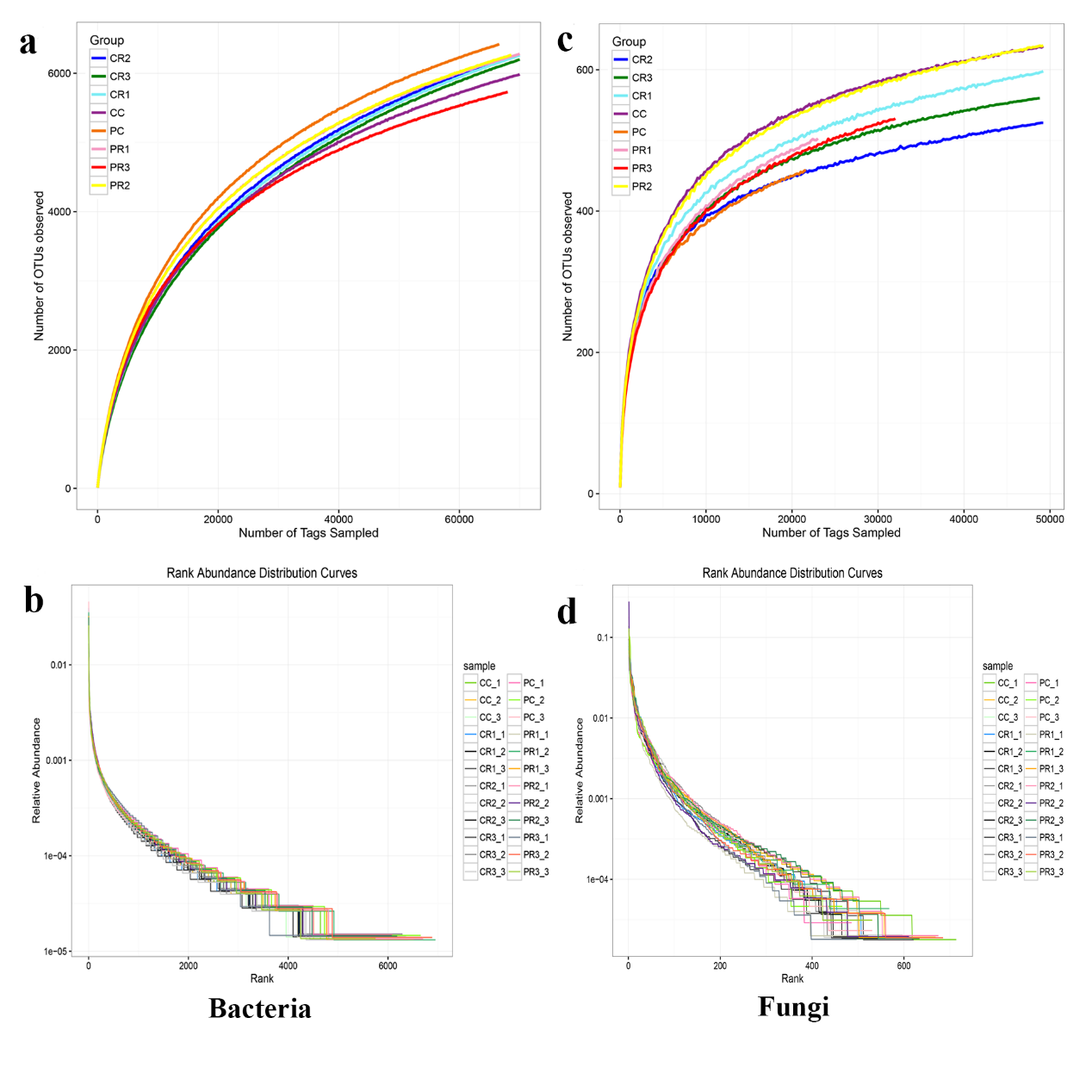


**Figure S2.** Effects of crop rotation in different years on microbial diversity in peanut rhizosphere soil: (a) rarefaction curves of bacteria; (b) rank abundance curve of bacteria; (c) rarefaction curves of fungi; (d) rank abundance curve of fungi.


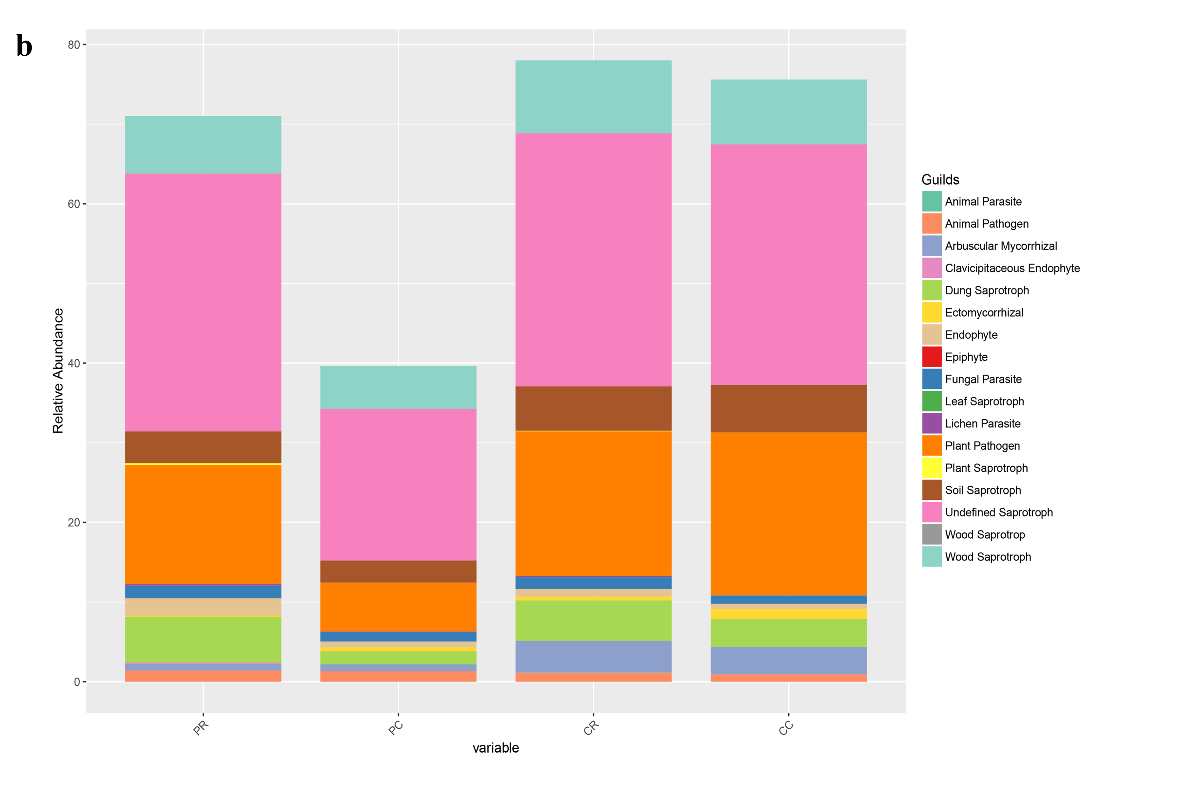

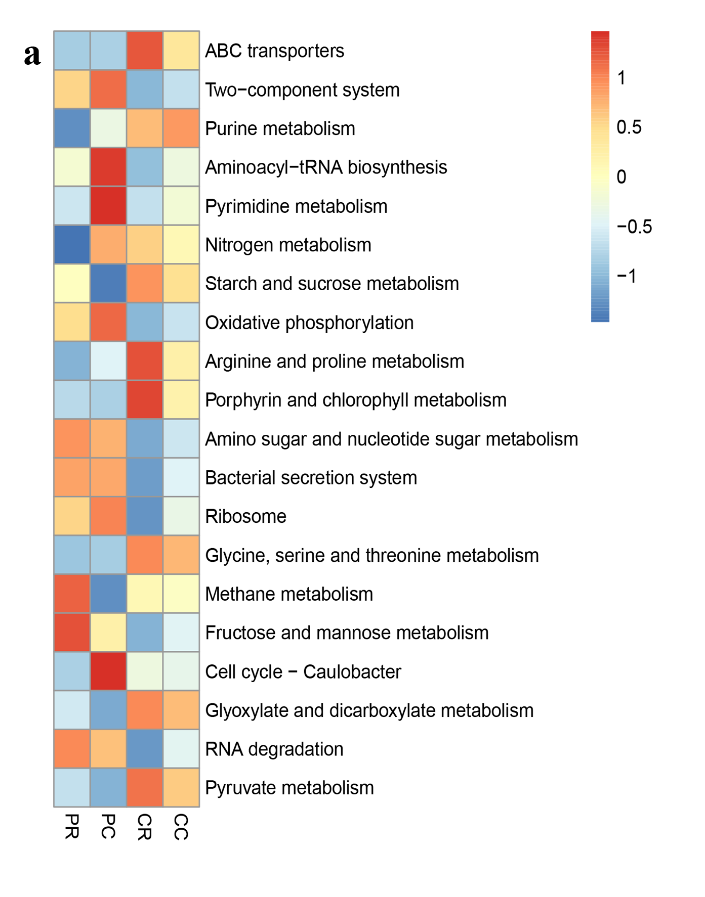


**Figure S3.** Effects of crop rotation in different years on microbial functional in peanut rhizosphere soil: (a) bacteria; (b) fungi.

**Table S1.** Relative abundances in bacterial and fungal communities.

|  |  | **PR** | **PC** | **CR** | **CC** |
| --- | --- | --- | --- | --- | --- |
| **Bacteria** | **Unclassified** | 1.331 | 1.8 | 1.235333333 | 1.124 |
|  | **Others** | 4.308766667 | 5.7334 | 3.808566667 | 3.8809 |
|  | **Bacteroidetes** | 1.4376 | 1.1824 | 1.991 | 1.9325 |
|  | **Gemmatimonadetes** | 3.250533333 | 4.208 | 4.150566667 | 4.2716 |
|  | **Firmicutes** | 3.9052 | 4.422 | 4.059166667 | 4.1011 |
|  | **Verrucomicrobia** | 6.713333333 | 6.4433 | 3.923633333 | 3.7135 |
|  | **Chloroflexi** | 7.342066667 | 8.089 | 8.074633333 | 7.502 |
|  | **Acidobacteria** | 11.60106667 | 11.0787 | 8.504233333 | 7.8124 |
|  | **Planctomycetes** | 22.9352 | 18.4487 | 11.79593333 | 15.6414 |
|  | **Proteobacteria** | 19.92786667 | 20.4874 | 23.73906667 | 23.9369 |
|  | **Actinobacteria** | 17.24743333 | 18.1071 | 28.7178 | 26.0839 |
| **Fungi** | **Unclassified** | 5.128333333 | 18.485 | 5.336 | 4.316 |
|  | **Others** | 0.2474 | 0.4952 | 0.209466667 | 0.1687 |
|  | **Chytridiomycota** | 0.752166667 | 2.327 | 1.093266667 | 3.2721 |
|  | **Glomeromycota** | 0.962966667 | 1.402 | 3.938133333 | 3.5295 |
|  | **Mortierellomycota** | 4.764733333 | 3.4503 | 3.393666667 | 6.0607 |
|  | **Basidiomycota** | 3.906933333 | 5.1019 | 6.725866667 | 13.3121 |
|  | **Ascomycota** | 84.23763333 | 68.7383 | 79.30376667 | 69.3408 |

Note: PR: rotation peanut, PC: continuous peanut, CR: rotation maize, CC: continuous maize.
